# Multi-Omics Analysis Reveals a Correlation between the Host Phylogeny, Gut Microbiota and Metabolite Profiles in Cyprinid Fishes

**DOI:** 10.3389/fmicb.2017.00454

**Published:** 2017-03-17

**Authors:** Tongtong Li, Meng Long, Huan Li, François-Joël Gatesoupe, Xujie Zhang, Qianqian Zhang, Dongyue Feng, Aihua Li

**Affiliations:** ^1^State Key Laboratory of Freshwater Ecology and Biotechnology, Institute of Hydrobiology, Chinese Academy of SciencesWuhan, China; ^2^Key Laboratory of Environmental and Applied Microbiology, and Environmental Microbiology Key Laboratory of Sichuan Province, Chengdu Institute of Biology, Chinese Academy of SciencesChengdu, China; ^3^Nutrition, Métabolisme et Aquaculture, Institut National de la Recherche Agronomique, University of Pau and Pays de l’AdourSaint-Pée-sur-Nivelle, France; ^4^College of Fisheries and Life Science, Shanghai Ocean UniversityShanghai, China; ^5^Freshwater Aquaculture Collaborative Innovation Center of Hubei Province, Huazhong Agricultural UniversityWuhan, China; ^6^National Fisheries Technical Extension Center, Ministry of AgricultureBeijing, China

**Keywords:** correlation, host phylogeny, gut microbiota, metabolite profiles, cyprinid fishes

## Abstract

Gut microbiota play key roles in host nutrition and metabolism. However, little is known about the relationship between host genetics, gut microbiota and metabolic profiles. Here, we used high-throughput sequencing and gas chromatography/mass spectrometry approaches to characterize the microbiota composition and the metabolite profiles in the gut of five cyprinid fish species with three different feeding habits raised under identical husbandry conditions. Our results showed that host species and feeding habits significantly affect not only gut microbiota composition but also metabolite profiles (ANOSIM, *p* ≤ 0.05). Mantel test demonstrated that host phylogeny, gut microbiota, and metabolite profiles were significantly related to each other (*p* ≤ 0.05). Additionally, the carps with the same feeding habits had more similarity in gut microbiota composition and metabolite profiles. Various metabolites were correlated positively with bacterial taxa involved in food degradation. Our results shed new light on the microbiome and metabolite profiles in the gut content of cyprinid fishes, and highlighted the correlations between host genotype, fish gut microbiome and putative functions, and gut metabolite profiles.

## Introduction

Vertebrates harbor vast and complex microbial communities that colonize their gastrointestinal tracts (Walter et al., 2011). As a result of this intimate relationship, the gut microbiome has become an integral part of the digestive system. The gut microbiota strongly influences fish health by stimulating the development of the intestinal epithelium and the immune system, and impeding pathogenic microorganisms to colonize the intestinal tract (Sugita et al., 1991; Flint et al., 2008; Ley et al., 2008b; Roeselers et al., 2011; Li T. et al., 2016). In the gastrointestinal tract, commensal bacteria can synthesize essential amino acids, vitamins and short-chain fatty acids (SCFAs), and contribute to feed efficiency, especially by degrading indigestible plant polysaccharides (Gill et al., 2006). Therefore, gut content and feces contain small molecules that are considered to result from co-metabolism or metabolic exchanges between microbes and host cells (Chen et al., 2012).

The composition of gut microbial communities is shaped by various internal and external factors, such as host genotype, diet, lifestyle, and surrounding environment (e.g., water temperature, salinity) (Nayak, 2010; Sullam et al., 2012). Previous studies of mammalian species revealed that their gut microbiota clustered according to diet rather than host phylogeny (Ley et al., 2008a; Muegge et al., 2011). It was also shown that identical twins had still significant differences in their gut microbiota, although they shared much higher similarity between gut microbiota structures than genetically unrelated married couples (Zoetendal et al., 2001). In addition, some scientists demonstrated that the phylogenetic relationships of hominids were completely consistent with those of gut microbiota (Ochman et al., 2010; Moeller et al., 2014). During evolution, changes in the composition of gut microbiota may lead to shifts in its functions, which may finally influence host nutrition and environmental adaptability (Amato, 2013). Comparative analysis among various hosts and their microbiota revealed that both diet and host phylogeny have driven the evolution of gut microbiota (Ley et al., 2008a). However, the main driving factor remains controversial. Thus, identifying shifts in gut microbiota composition and diversity over evolutionary timescales will be crucial to understanding how gut microbiota of cyprinid fishes is involved in evolution and environmental adaptation.

The concept of metabonomics was first defined as “the quantitative measurement of the dynamic multiparametric metabolic response of living systems to pathophysiological stimuli or genetic modification” (Nicholson et al., 1999; Nicholson and Wilson, 2003). Nowadays, metabonomics provides a systematic approach to characterize the metabolic phenotype, which results from a coordinated physiological response to various intrinsic and extrinsic parameters including environment, drugs, dietary, lifestyle, genetics, and microbiome (Rezzi et al., 2007; Li et al., 2008; Zhou et al., 2014). Recently, complementary metabonomic approaches have been employed for the biochemical characterization of metabolic changes triggered by gut microbiota, dietary variation, and stress interactions (Martin et al., 2008; Wikoffa et al., 2009; Vitali et al., 2010). Solid phase micro-extraction followed by gas chromatography and mass spectrometry (GC/MS) represents a novel method for studying metabolic profiles of biological samples, which was considered as the gold standard in metabonomics (Harrigan and Goodacre, 2012). With this method, the volatile compounds, or those that can be made volatile, or stabilized by derivatization, are separated by gas chromatography and then detected by mass spectrometry (Roessner et al., 2000; Vernocchi et al., 2012, 2016). This approach has been increasingly applied to humans and other terrestrial vertebrates (Garner et al., 2007; Vitali et al., 2010; Antharam et al., 2016; Faber et al., 2016). However, studies on fish gut metabolome are scarce compared with those on terrestrial vertebrates using GC/MS. Global metabolite profiling performed here in on fish gut samples provided insights into the relationship between microbial populations and metabolites.

The polyculture of carps with different feeding habits is a traditional method to optimize the use of trophic resources in ponds (Li et al., 2015). In the present study, the intestinal microbial community structure and global metabolite profiles of five cyprinid fish species cohabitated in the same environment were investigated: herbivorous grass carp (*Ctenopharyngodon idellus*, HG) and blunt snout bream (*Megalobrama amblycephala*, HB); omnivorous crucian carp (*Carassius auratus*, OC); filter-feeding silver carp (*Hypophthalmichthys molitrix*, FS) and bighead carp (*Hypophthalmichthys nobilis*, FB). These five freshwater fishes are the major carps in Chinese aquaculture and widely cultivated for food. In 2012, the production of these species reached 14.48 million tons in China, accounting for about 62.02% of the total freshwater-cultured fish annual output (MoA, 2012). Furthermore, understanding the microbial community and metabolite profile in the gut of these fish species can provide useful information to improve health and productivity of these commercially valuable freshwater species.

To date, many studies focused on fish gastrointestinal microbiota, and such studies only concentrated on some factors (e.g., diet, feed habits, and genotype of host) that may affect fish gastrointestinal microbiota (Wu et al., 2012; Li et al., 2013, 2015; Ye et al., 2013). Little is known about the relationship among host evolutionary distance, gut microbiota and metabolic profiles in cyprinid fishes. Here, we investigated two key questions. First, do host genotype, gut microbiota and gut metabolic profiles correlate between them? Secondly, what is the relationship between dominant microbes and key metabolic products in the fish guts?

## Materials and Methods

### Sample Collection and Pyrosequencing

All fish samples were harvested from Dongxihu Fish Farm, Wuhan City, Hubei Province, China, on April 10th, 2012, when the water temperature in the pond was 18°C. The adult cyprinid fishes were harvested from the same earth pond of approximately 0.5 ha. The fish were fed with commercial diet from JiuZhou Shennong Pharmaceutical Co., Ltd., Wuhan, China (crude protein ≥ 30.0%, crude fiber ≥ 12.0%, crude ash ≤ 15.0%, calcium = 0.4–2.5%, phosphate ≥ 0.7%, salt = 0.3–1.2%, moisture ≤ 12.5%, and lysine ≥ 1.2%). The five fish species with different feeding habits were as follows: herbivorous grass carp (*Ctenopharyngodon idellus*, HG) and blunt snout bream (*Megalobrama amblycephala*, HB); omnivorous crucian carp (*Carassius auratus*, OC); filter-feeding silver carp (*Hypophthalmichthys molitrix*, FS) and bighead carp (*Hypophthalmichthys nobilis*, FB). Three healthy individuals of each species were randomly harvested using nets. The average body weights were c. 1.2, 0.5, 0.3, 1.0, and 1.0 kg for grass carp, blunt snout bream, crucian carp, silver carp, and bighead carp, respectively. The fish were carried on ice to the laboratory within 2 h. Prior to dissection, all fish were euthanized with an overdose of MS 222 (Sigma, Germany). All procedures for handling and euthanasia of fish were conducted as described by Wu et al. (2012). The intestines were aseptically removed from the abdominal cavity under sterile environments and the contents of hindgut (lower one-third of the full intestine) from the three individuals of each species were gently squeezed out, placed in a sterile tube and stored at -80°C. The methods used in this study were reviewed and approved by the ethics committee of the Institute of Hydrobiology, Chinese Academy of Sciences, and were carried out in accordance with the relevant guidelines, including any relevant details (Approval ID: keshuizhuan 08529).

DNA preparation, PCR amplification, and pyrosequencing were performed as described by Li et al. (2015). Briefly, total DNA was extracted from 250 mg of contents by using E.Z.N.A. Stool DNA kit (OMEGA, Bio-Tek, USA). A primer set (515F/926R) was used to amplify the V4 region of the 16S rRNA gene for analyzing gut microbiota. Each sample was amplified in triplicate in a reaction volume of 20 μL containing 1 × Ex Taq PCR buffer, 10 pM of each primer, 1.25 U Takara Ex Taq (all TaKaRa Biotechnology Co., Ltd., Dalian, China), and 5 ng genomic DNA using the following program: 5 min at 95°C, followed by 30 cycles of 94°C for 30 s, 56°C for 30 s, and 72°C for 30 s, and finally, 10 min at 72°C. PCR products were purified using the QIAEX II Gel Extraction Kit (QIAGEN). Library preparation and pyrosequencing was performed at the Chinese National Human Genome Center in Shanghai with 454 GS FLX sequencing platform (Roche). The raw sequences are available through the NCBI/EBI/DDBJ Sequence Read Archive (Accession No. DRA002627 and DRA001264^1^).

### Phylogenetic Analysis of Fishes

Phylogenetic analysis of five cyprinid species (grass carp, blunt snout bream, crucian carp, silver carp, and bighead carp) were constructed from two mitochondrial genes (*COI* and *cytb*) using MEGA program (version 6.0) (Tamura et al., 2013). The corresponding gene sequences were downloaded from GenBank (accession numbers in Supplementary Table S1). Common rabbits (*Oryctolagus cuniculus*) was used as the outgroup taxon. The concatenated sequences of the two mitochondrial genes were aligned using ClustalW in MEGA (pairwise and multiple alignment parameters: gap opening penalty 15, gap extension penalty 6.66, delay divergent sequences 30%, DNA transition weight 0.5, and no use of a negative matrix) (Thompson et al., 1994; Tamura et al., 2013). The phylogenetic tree was constructed based on the aligned DNA sequences by the neighbor-joining method using Kimura 2-parameter model in MEGA (Test of phylogeny options: Bootstrap 999 replicates; Substitutions to include: Transitions + Transversions; Rates among sites: Uniform rates; Pattern among lineages: Same (Homogeneous); Gaps/Missing Data: Complete Deletion; Codon Positions: 1st + 2nd + 3rd + Non-coding) (Kimura, 1980; Tamura et al., 2013). The identical triplicates of the concatenated sequences were used to generate the phylogenetic distance matrix for Mantel tests in MEGA using the same methods as described above.

### Metabolite Extraction and Metabolite Profiling Analysis

Extraction of metabolites from gut content samples was according to Lisec et al. (2006) with some modification. Gas chromatography was performed on a HP-5MS capillary column [5% phenyl/95% methylpolysiloxane (30 m × 250 μm i.d., 0.25 μm film thickness, Agilent J&W Scientific, Folsom, CA, USA)] to separate the derivatives at a constant flow of 1 mL/min helium. Raw gas chromatography/mass spectrometry data (GC/MS) data were converted into CDF format (NetCDF) files by Agilent GC/MS 5975. Data Analysis was processed by the XCMS^2^. Detailed protocols for metabolite extraction and GC/MS analyses were provided in Supporting Information Methods.

### Pyrosequencing Data Processing and Bioinformatics Analysis

Quality filtering, denoising, and chimera checking of the sequences obtained from pyrosequencing were performed with the mothur software (Schloss et al., 2009), as described previously (Li et al., 2015). The average read length was 250 bp. The resulting sequences were rarefied separately by random subsampling of 5,935 sequences from each sample in mothur, thereby equalizing the sampling effort across samples (Schloss et al., 2009). To predict the functional profiles of microbial communities, OTUs were picked using a closed reference (Greengenes ver. 13.5) at 97% sequence similarity, with normalization to control for differences in 16S rRNA copy number among OTUs, and their metagenomic contributions were predicted using PICRUSt, from the Kyoto Encyclopedia of Genes and Genomes (KEGG) pathways (DeSantis et al., 2006; Kanehisa et al., 2012; Langille et al., 2013). The pertinence of the metagenome predictions was assessed by computing NSTI (Nearest Sequenced Taxon Index).

Non-metric Multidimensional Scaling (NMDS) plots based on Bray–Curtis distance were used to visualize the separation of microbiota structure, metabolic profiles, and presumptive functions across different groups. Statistical testing among variation in microbial community composition was carried out using the analysis of similarity (ANOSIM). Mantel tests based on Bray–Curtis distance and Spearman’s correlation analysis were applied to evaluate the correlations between gut microbial dissimilarity, metabolic profiles and host evolutionary distance. Differences between groups were evaluated by One-way Analysis of Variance (One-way ANOVA) followed by Tukey’s *post hoc* test. The heatmap was constructed using the heatmap 2 function of the R gplots package (Gentleman et al., 2004). The machine learning algorithm Random Forests (Breiman, 2001) was used to identify the discriminatory OTUs and presumptive functions between species using the package R randomForest with 5000 trees and all default settings. The Boruta algorithm was used to select the features with predictive power (Kursa and Rudnicki, 2010). In order to understand the relationship between some specific gut microbes and certain metabolites, redundancy analysis (RDA) was performed. All statistical analyses were performed using R version 3.1.0. All permutation tests (i.e., ANOSIM and Mantel test) were conducted using 999 permutations.

## Results

### Differences in Bacterial Community Diversity and Metabolite Profiles

The completeness of sampling was estimated with Good’s coverage, by calculating the probability that a randomly selected amplicon sequence was already detected in the same sample (**Table 1**). The coverage ranged from 93.29 to 99.63% (97.04 ± 2.64 %), indicating that between 15 and 270 [1/(1-‘Good’s coverage’)] additional reads would need to be sequenced before detecting a new OTU. This level of coverage suggested that the majority of bacterial OTUs present in the samples were identified in this study.

**Table 1:** Number of sequences analyzed, observed diversity richness (OTUs), and estimated sample coverage for 16S rRNA gene libraries of the different samples.

Samples	No. sequences	OTUs	Coverage (%)
FB1	5935	835	93.36
FB2	5935	427	96.41
FB3	5935	761	94.00
FS1	5935	913	93.55
FS2	5935	793	93.68
FS3	5935	790	93.29
HB1	5935	113	99.21
HB2	5935	159	98.57
HB3	5935	201	98.23
HG1	5935	143	98.75
HG2	5935	153	98.92
HG3	5935	124	99.26
OC1	5935	90	99.22
OC2	5935	60	99.63
OC3	5935	55	99.51


*FB1–FB3 for bighead carp, FS1–FS3 for silver carp, HB1–HB3 for blunt snout bream, HG1–HG3 for grass carp, and OC1–OC3 for crucian carp.*

The gut microbiota of filter-feeding silver carp and bighead carp samples yielded the significantly higher alpha-diversity indices (ANOVA, *p* < 0.05), followed by those of herbivorous grass carp, blunt snout bream, and omnivorous crucian carp (**Table 1** and Supplementary Figure S1).

**FIGURE 1:**
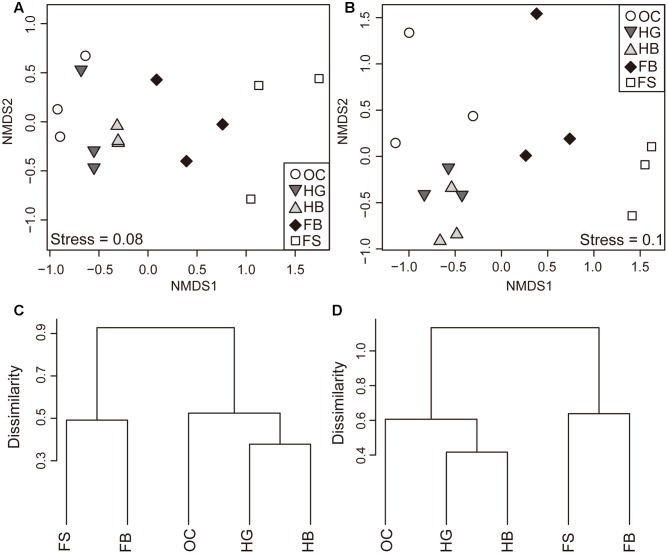
**Differences in the composition of gut microbial and metabolite profiles.** Non-metric Multidimensional Scaling (NMDS) plot showing variation in the composition (Bray–Curtis distance) of gut bacterial communities **(A)** and metabolite profiles **(B)** among different fish species. Hierarchical clustering of sample groups based on the mean relative abundance of each OTU **(C)** and metabolite **(D)** in each fish species. Abbreviations: FS, silver carp; FB, bighead carp; HG, grass carp; HB, blunt snout bream; OC, crucian carp.

Similarities of the bacterial communities and metabolite profiles between samples were compared by ANOSIM and NMDS based on Bray–Curtis (Singh et al., 2015). ANOSIM revealed significant differences in the structure (**Figure 1A**; ANOSIM, *r* = 0.59, *p* = 0.001) of gut microbiota among different fish species. There was a tendency for the bacterial profiles of fish to separate by feeding habits (ANOSIM, *r* = 0.60, *p* = 0.001). In addition to differences in bacterial communities, the five species of fish had also marked differences in metabolite profiles (**Figure 1B**; ANOSIM, *r* = 0.60, *p* = 0.001). There was a consistent effect of feeding habits on the metabolite profiles of fish (ANOSIM, *r* = 0.74, *p* = 0.001).

Moreover, the distances of the bacterial communities among the individuals between different feeding habits were calculated (Supplementary Figure S2). We found that distances between filter-feeding and herbivorous carps (fil vs. her), as well as between filter-feeding and omnivorous carps (fil vs. omn) were significantly higher as compared with those between herbivorous and omnivorous species (her vs. omn) (Supplementary Figure S2A; ANOVA, *p* < 0.05). For the herbivorous and omnivorous species, the distances among the individuals within the same feeding habits (HG vs. HB) were significantly lower than those in different feeding habits (HG vs. OC and HB vs. OC) (Supplementary Figure S2B; ANOVA, *p* < 0.05). Similar results were observed for the profiles of metabolites (Supplementary Figures S2C,D). The filter-feeding carps displayed greater within-group variability in gut microbiota and metabolic profiles, compared to the herbivorous species (Supplementary Figures S3A,B).

The NMDS plot indicated that the gut bacterial communities in each species clustered firstly together, and then they tend to cluster with those fish species within the same feeding habits (**Figures 1A,B**). Similar clusters were observed for metabolite profiling (**Figures 1C,D**).

### Variation in Gut Microbiota Composition, Gut Metabolites and Evolutionary Distance

**FIGURE 2:**
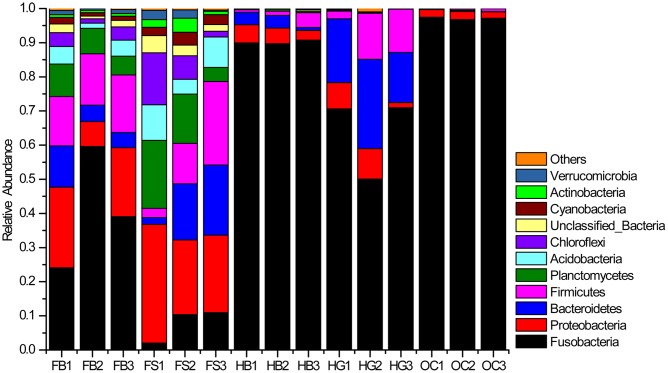
**Bacterial composition of the different communities (relative read abundance of bacterial phyla within each community).** Lanes FB1–FB3, FS1–FS3, HB1–HB3, HG1–HG3, and OC1–OC3 correspond to the three individuals of bighead carp, silver carp, blunt snout bream, grass carp, and crucian carp, respectively.

The abundances of 10 major phyla were commonly observed across all the samples (**Figure 2**). Those sequences that were not assigned to known microbial phyla were designated as “Unclassified Bacteria,” which represented 1.08% of the entire data set. A few phyla occurred at low abundance and sporadically in some samples were referred to as “Others” (˜0.24% of the total sequences). Fusobacteria was the most dominant phylum in the gut bacterial communities, representing 59.96% of the total sequences. This phylum was the highly dominant, especially in the gut samples of omnivorous crucian carp (OC1–OC3), followed by herbivorous grass carp (HG1HG3) and blunt snout bream (HB1–HB3), and then filter-feeding bighead carp (FB1–FB3) and silver carp (FS1–FS3). Proteobacteria and Planctomycetes were two major phyla of the gut content samples in filter-feeding silver carp and bighead carp. Bacteroidetes and Firmicutes were also the dominant phyla, except in crucian carp.

**FIGURE 3:**
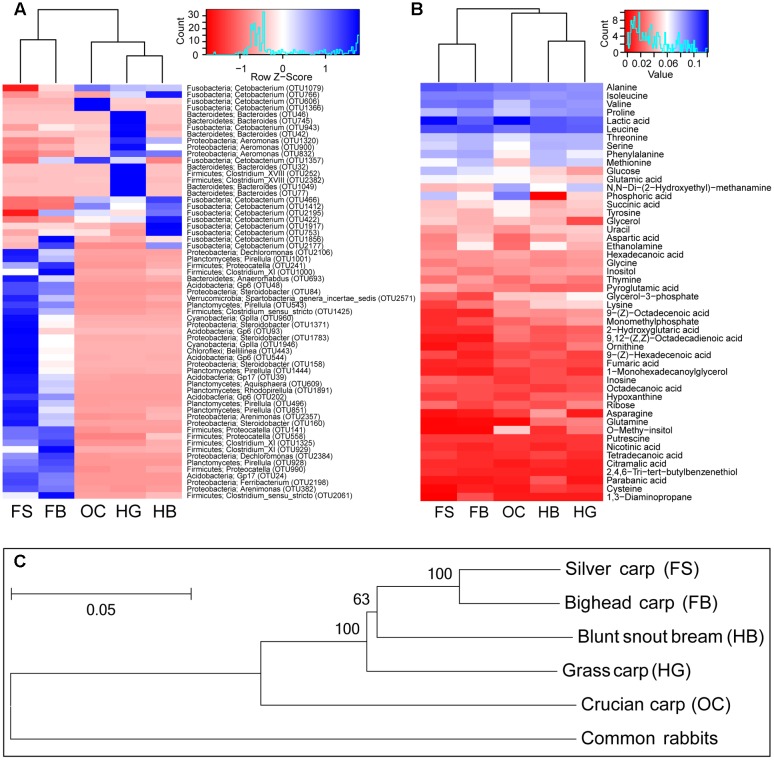
**Summary of the variation in gut microbiota, gut metabolite, and evolutionary distance among different fish species.** **(A)** A heat map of the mean abundances of the prominent OTUs (average abundance > 0.1%) assigned to genus level among different fish species. The rows represent the 63 predominant bacterial OTUs, and the values in the heatmap represent the Z-transformed relative percentage of each OTU. Phylum and genus level classifications of OTUs (OTU ID in parentheses) are noted also. **(B)** A heat map of the mean percent abundances of top50 metabolite among different fish species. Both heat map based on the same hierarchical clustering solution (Bray–Curtis distance metric and complete clustering method). **(C)** Phylogenetic tree of the five cyprinid fish species based on the concatenated sequences of the two mitochondrial genes. The numbers on the nodes are neighbor-joining bootstrap values (values > 50 are shown), and bootstrap was replicated 999 times. The bar shows the relative branch length distance computed using the Kimura 2-parameter method. HG, grass carp; HB, blunt snout bream; OC, crucian carp; FS, silver carp; and FB, bighead carp.

The two most predominant bacteria in the gut microbiota of crucian carp were two OTUs assigned to genera *Cetobacterium* (97.09% ± 0.36%) and *Aeromonas* (1.45% ± 0.83%) in the phyla Fusobacteria and Proteobacteria, respectively (**Figure 3A**). The genus *Cetobacterium* was also abundant in the gut content of the other fishes: in herbivorous blunt snout bream (89.87% ± 0.66%) and grass carp (63.80% ± 12.02%); in filter-feeding bighead carp (40.68% ± 17.63%) and silver carp (7.64% ± 4.90%). In grass carp, a large proportion of OTUs belonging to the genus *Bacteroides* (19.11% ± 5.96%), followed by the genera *Aeromonas* (5.13% ± 3.72%), *Clostridium* XVIII (1.92% ± 1.18%), *Clostridium* XlVb (0.80% ± 0.63%), and *Proteocatella* (0.71% ± 0.39%). OTUs belonging to *Aeromonas* (2.86% ± 2.06%), *Clostridium* XI (0.34% ± 0.25%), *Proteocatella* (0.28% ± 0.38%), and *Pirellula* (0.23% ± 0.07%) were also common in blunt snout bream. Other genera were relatively abundant in the gut microbiota of silver carp and bighead carp, namely: *Pirellula*, Gp6, *Anaerorhabdus*, *Steroidobacter*, *Proteocatella*, *Dechloromonas*, GpIIa, *Clostridium* XI and *Clostridium sensu stricto* (cluster I). In the gut content of the two filter-feeding species, Acidobacteria clade Gp6 and Cyanobacteria clade GpIIa were also abundant (**Figure 3A**).

**FIGURE 4:**
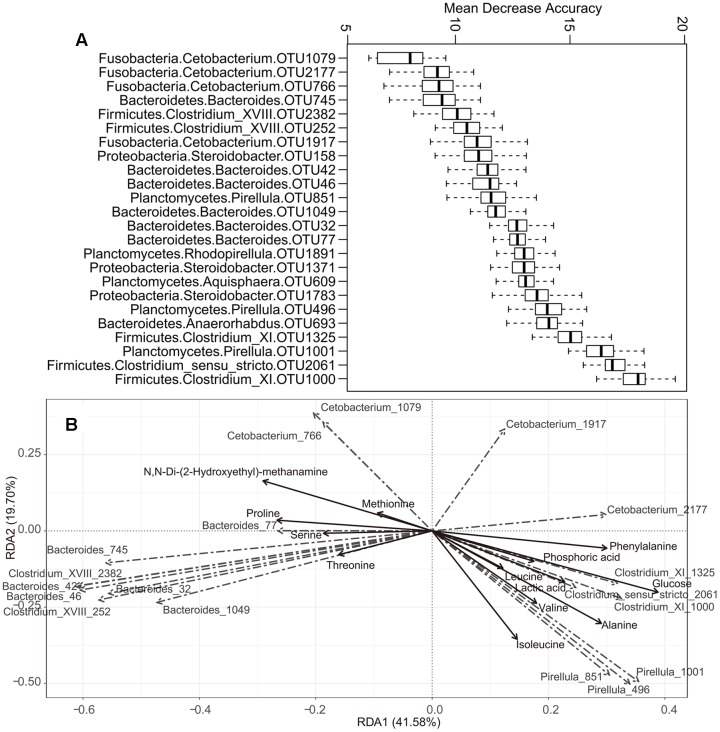
**The correlation between some specific microbes and metabolite.** **(A)** Top 24 OTUs that differentiate among different fish species as revealed by Random Forest. The phylum and genus classifications are also provided. The OTU ID is given after the genus. **(B)** Redundancy analysis (RDA) of some specific gut microbes responding to metabolite. Only those metabolite with relative abundances > 3% were shown.

The prominent OTUs (mentioned in **Figure 3A**) used as inputs in the Random-Forest model paired with Boruta feature selection. An importance score, the mean decrease accuracy (MDA) was assigned to each OTU, based on the error increase corresponding to the removal of this feature from the predictors. A total of 24 significant OTUs were selected based on Boruta algorithm. The importance score (MDA) of these OTUs was illustrated in **Figure 4A**. Interestingly, the relative abundance of OTUs belonging to *Pirellula*, *Bacteroides*, *Clostridium* (cluster I, XI, and XVIII), and *Cetobacterium* were among the most discriminative features between fish species.

In this investigation, 99 different metabolites were detected in the gut content samples by means of GC/MS analysis. The metabolite profiles of gut contents varied also among different fish species, mainly due to lactic acid and some amino acids. The gut samples of cyprinid fishes, especially crucian carp and silver carp, had a high percentage of lactic acid (**Figure 3B**). The gut samples of crucian carp had higher percentages of phosphoric acid, whereas the gut samples of filter-feeding carps had higher percentages of leucine, followed by herbivorous carps. Other dominant metabolites corresponded to alanine, isoleucine, and valine, which were more abundant in the filter-feeding species.

The phylogenetic structure inferred here was consistent with that of previous studies (**Figure 3C**) (Kong et al., 2007; Tao et al., 2010; Li et al., 2012). The results showed that silver carp and bighead carp were the most closely related species (**Figure 3C**), and the two filter-feeding species shared more diverse gut bacteria, such as OTUs of *Proteocatella*, *Clostridium* XI and *Clostridium sensu stricto*, and metabolites, such as leucine, alanine, isoleucine, and valine (**Figures 3A,B**). Among herbivorous and omnivorous carps, more closely related species tended to harbor more similar gut microbiota composition and metabolite profiles (**Figures 3A–C**). OTUs belonging to *Aeromonas*, *Clostridium* XI, *Proteocatella*, *Pirellul*a and relatively high percentages of leucine were common in both grass carp and blunt snout bream, while, *Cetobacterium*, lactic acid and phosphoric acid were more abundant in crucian carp.

### The Relationship between Evolutionary Distance, Gut Microbiota and Metabolite Profiles

Mantel tests were performed to detect the correlations between the fish evolutionary distance, gut microbiota and metabolite profiles. In particular, we found that the evolutionary distance was positively associated with the dissimilarity of gut microbiota (Mantel test, *r* = 0.34, *p* = 0.009) and that of metabolite profiles (Mantel test, *r* = 0.36, *p* = 0.003). These correlations were relatively low, but significant. The gut bacterial and metabolite profiles were also significantly related (Mantel test, *r* = 0.61, *p* = 0.001).

The correlation between some specific gut microbes and metabolites was detected using RDA (**Figure 4B**). For example, the OTUs assigned to *Pirellula* were positively correlated with isoleucine, valine, leucine, and alanine, while OTUs belonging to *Clostridium* (cluster I and XI) showed positive correlations with lactic acid, glucose, and phosphoric acid. In addition, OTUs of *Bacteroides* and *Clostridium* XVIII correlated positively with proline, serine, and threonine. *Cetobacterium* OTU1079 and OTU766 correlated positively with methionine and N,N-Di-(2-Hydroxyethyl)-methanamine, while OTU2177 in the same genus was positively correlated with phenylalanine.

The presumptive functions of the gut microbial communities were also examined using PICRUSt. The fish gut samples had NSTI values of 0.11 ± 0.05. For comparison, Langille et al. (2013) found that Human Microbiome Project samples had the lowest (best) NSTI values (0.03 ± 0.2). Other mammalian guts had a higher mean NSTI value (0.14 ± 0.06), and diverse communities such as soil also had a much higher NSTI value (0.17 ± 0.02). Thus, the fish gut samples provide a reasonable data set to examine predictions from PICRUSt. We found there were significant differences in predicted microbial functions across species.

A total of 29 significant level 3 KEGG Orthology (KO) functions were selected based on Boruta algorithm. The majority of the most discriminative putative functions were in the category of metabolism (Supplementary Figure S4A). In this context, the most noteworthy functional genes were involved in carbohydrate metabolism (butanoate metabolism, citrate cycle, glycolysis/gluconeogenesis, and pyruvate metabolism), amino acid metabolism (arginine and proline metabolism, tyrosine metabolism and selenocompound metabolism), energy metabolism (nitrogen metabolism), lipid metabolism (glycerolipid metabolism), metabolism of cofactors and vitamins (porphyrin and chlorophyll metabolism), and xenobiotics biodegradation and metabolism (chloroalkane and chloroalkene degradation). The result indicated that these categories were particularly important in differentiating the putative functions of gut microbiota among fish species. The distribution of points representing the inferred function of gut microbiota suggested that fishes within the same feeding habits had more similarity in the predicted functions of the microbiome than fishes with different feeding habits (Supplementary Figure S4B).

## Discussion

To our knowledge, the present study was the first one that addressed the relationship between host phylogeny, gut microbiota and metabolic profiles in cyprinid fishes raised under identical husbandry conditions. Our results showed (1) the presence of specific fermentative bacteria populations in different cyprinid species; (2) the correlations between host evolutionary distance, gut microbiota and metabolic profiles of cyprinid fishes; (3) the influence of feeding habits and fish species on gut microbial and metabolic profiles. Li et al. (2014) explored the gastrointestinal microbiota of eight fish species with different feeding habits, but these wild fishes were not raised under identical husbandry conditions. In particular, the differences of gut microbiota composition between closely related fish species were not clearly defined, and the specificity as an adaptive measure remains undetermined. Our results shed new light on the microbiota of cyprinid fishes and highlighted the correlations between host genotype, gut microbiota and presumptive functions, and gut metabolite profile. Furthermore, understanding the microbial community and metabolite profile in the guts of these fish species can provide useful information to improve the health and productivity of these commercially valuable freshwater species.

### The Composition of Gut Microbial and Metabolite Profiles Differed

The filter-feeding carps, which have the smallest phylogenetic distance between them, displayed higher alpha-diversity and greater intragroup variability, compared with the other dietary groups (Supplementary Figures S1, S3). An explanation may be ascribed to feeding behavior, although feed pellets were supplied in the pond. In general, diet-associated microbes have a wider diversity when they originate from diverse feeds, and the animals that consume more diverse feeds may be exposed to carry more diverse microbes (Laparra and Sanz, 2010). Compared with other species, the filter-feeding carps actively swim all around and expand a variety of feed items by filtering large volumes of water (Fang et al., 2013; Zhang and Kang, 2013). Such behavior can increase the variability in the composition of feed intake. In fact, it has been shown that diet diversity was associated with the variation of gut microbiota composition (Li H. et al., 2016). This filter-feeding activity resulted likely in more diverse microbes and greater variation of microbiota composition in filter-feeding carps (Verschuere et al., 2000; Tetlock et al., 2012; Li H. et al., 2016).

The environment affects the gut microbiota of fish and mammals (Ley et al., 2008a; Wu et al., 2012), but the same environment in the present study did not result in similar gut microbiota and metabolite profiles among the five carp species. Our results demonstrated that host species could affect not only gut microbiota, but also metabolite profiles (**Figure 1**). Recent studies showed that some particular genes may influence host immunity or physiology, and control the size of individual microbial populations (Benson et al., 2010; Bolnick et al., 2014). However, diet may also influence the phylogenetic relationship of gut microbiota among hosts. In our study, the natural feeding habits among the five carp species differed, and their feed intake was likely different, even though commercial food was supplied in the pond. As most animals lack the ability to degrade and to digest cellulose, however, certain species are capable of digesting cellulose because of their gut microbiota (Poulsen et al., 2014; Wang et al., 2015). Plant polysaccharide-degrading bacteria are particularly important for food degradation in the gut of herbivorous carps, namely grass carp and blunt snout bream (Wu et al., 2012, 2015). Contrastingly, filter-feeding activity resulted in a much higher bacterial diversity in the gut of silver carp and bighead carp, compared to those of the other species (Verschuere et al., 2000; Tetlock et al., 2012). Thus, the diet may also explain the differences of phylogenetic relationship in host-gut microbiota.

As the feeding habits similarity was related to the phylogenetic distance between fishes (**Figure 3C**), it was not possible to differentiate the relative influence of the diet from that of genetic factors, when studying the gut microbial composition and metabolite profiles in this study. Future interspecific studies with well-defined diets are needed for addressing the issue.

### Correlation between Host Genetics, Gut Microbiota and Metabolic Profiles of Cyprinid Fishes

Additionally, the carps with the same feeding habits had more similarity in gut microbiota composition and metabolite profiles (**Figures 3A,B**). This similarity among closely related host species suggested some degree of correlations between hosts and microbes, and further analysis showed the positive correlations between evolutionary distance and gut microbiota or metabolite profiles. In vertebrates, closely related host lineages harbor more similar gut microbiota than distantly related lineages (Ochman et al., 2010; Sanders et al., 2014). *Bacteroides*, *Aeromonas*, *Clostridium* XVIII, *Clostridium* XlVb, *Clostridium* XI, and *Pirellula* were the dominant genera identified in the herbivorous carps (**Figure 3A**). Some strains of these genera are associated with plant-rich diets, and widely known as cellulose-degrading bacteria, which are particularly important for food degradation in the gut of herbivorous carps (Hayashi et al., 2002; Flint et al., 2008; King et al., 2012; Wu et al., 2012; Ziemer, 2013; Li et al., 2015). The two filter-feeding species shared more diverse dominant gut bacteria (**Figure 3A**). Some members of these bacterial communities included not only species with saccharolytic and fiber-fermenting activities, but also proteolytic species, such as *Proteocatella*, *Clostridium* XI and *Clostridium sensu stricto* (Lubbs et al., 2009; Pikuta et al., 2009; Schwab et al., 2011). Our results are consistent with another convincing study, suggesting that differences in the gut microbiota of closely related stickleback populations were correlated with host genetic divergence, even after controlling for food and water microbes (Smith et al., 2015). This host genetic control may explain the conserved composition of the gut microbiota with closely related species. By exerting top-town selection pressure, host genetic control may overcome microbial competition within the gut ecosystem and promote microbes that benefit the host (Benson et al., 2010). However, a correlation with host phylogeny by itself does not allow to discern whether genetic factors are acting through the direct physiological control of gut microbiota by the host (Benson et al., 2010; Bolnick et al., 2014; Thaiss et al., 2014), or whether the correlation was due to the dietary strategy of the species (Ley et al., 2008a; Muegge et al., 2011).

The metabolic importance of gut microbiota was illustrated by the fact that genetically homogeneous fishes may have diverse metabolic profiles when they have structurally different gut microbiota. The gut bacteria in carps belong to clades of fermentative bacteria, whose metabolism may yield varying amounts of products (Ni et al., 2014; Wu et al., 2015). These differences suggest that variation in the structure of gut bacterial communities should result in variation in the structure of gut metabolic profiles. Here we found that the gut bacterial and metabolic profiles of cyprinid fishes was significantly related (Mantel test, *r* = 0.61, *p* = 0.001; **Figures 3A,B**). The discriminative OTUs between different fish species (**Figure 4B**) included members of *Pirellul*a, *Bacteroides*, *Clostridium* (cluster I, XI, and XVIII), and *Cetobacterium*. Most of these genera are involved in food degradation, as described above (Lubbs et al., 2009; Schwab et al., 2011; King et al., 2012; Ziemer, 2013). A difference in abundance of metabolites suggested a difference in the ability of food utilization by gut bacteria among cyprinid fishes. Due to the complex composition of gut microbiota, various metabolites were detected which correlated positively with various bacterial taxa (**Figure 4B**). Our results showed that fermentative subsets of gut bacterial taxa might be selectively stimulated by food (carbohydrates, proteins or lipids) and might therefore contribute to digestion, especially by degrading plant-derived polysaccharides in fish gut. However, this study disclosed the correlations between metabolism and gut microbiota, but not the causal relationships. In order to investigate the functional roles of gut microbiota, especially those associated with food degradation and digestive metabolism, a strict control of experimental conditions in laboratories should be applied, and multiple analytical approaches are necessary.

Lactic acid and SCFAs are among the dominant metabolites produced by commensal microbiota. Aldunate et al. (2015) suggested their potential use as biomarkers of disease and/or disease susceptibility, due to antimicrobial and immune modulatory properties. Interestingly, amino acids can serve as precursors for the synthesis of SCFA by bacteria, suggesting an interplay between microbial activity and host amino acid and SCFA homeostasis (Neis et al., 2015). The PICRUSt results suggested also that gut microbiota may exert physiological functions linked to amino acid metabolism and glycolysis (Supplementary Figure S4), which is consistent with another study about the gut microbiome of grass carp (Ni et al., 2014). However, these results still need further confirmation.

In summary, this is the first report that addressed the relationship between host phylogeny, gut microbiota and metabolic profiles in cyprinid fishes using next-generation sequencing and GC/MS. The results showed that host genotype, gut microbiota, and gut metabolite profile have concordant phylogenic relationship. However, only five fish species with a small sample size of three individuals per species were used to construct the relationships between host phylogeny, gut microbiota and metabolic profiles. The correlation scores presented in our study are between 0.3 and 0.4. This relatively low correlation coefficient may be due to insufficient sampling. More diverse host species and larger populations should be surveyed in the future work to confirm these results and extend the knowledge about the relationship among host evolutionary distance, gut microbiota and metabolic profiles in cyprinid fishes. In addition, it remains to be determined whether evolutionary distance is associated with gut microbial diversity or metabolic profiles in other host species.

## Author Contributions

TL, ML, DF, and AL conceived the research. TL, ML, HL, and XZ performed the experiments. TL wrote the manuscript. TL, HL, F-JG, and AL edited the manuscript. QZ, XZ, and ML contributed sampling, reagents or data analysis pipeline. All authors reviewed and accepted the manuscript.

## Conflict of Interest Statement

The authors declare that the research was conducted in the absence of any commercial or financial relationships that could be construed as a potential conflict of interest.

## Footnotes

1 http://trace.ddbj.nig.ac.jp/DRASearch/

2 www.bioconductor.org
