# Supplementary material for: Multi-Omics Analysis Reveals a Correlation between the Host Phylogeny, Gut Microbiota and Metabolite Profiles in Cyprinid Fishes
Source: Front Microbiol. 2017 Mar 17;8:454. doi: 10.3389/fmicb.2017.00454 (PMC5355437; doi:10.3389/fmicb.2017.00454)

Supplementary Material

**Multi-omics analysis reveals a correlation between the host phylogeny, gut microbiota and metabolite profiles in cyprinid fishes**

Tongtong Li^1,2§^, Meng Long^1§^, Huan Li^2^, François-Joël Gatesoupe^4^, Xujie Zhang^5^, Qianqian Zhang^1,6^, Dongyue Feng^3*^,Aihua Li^1,6*^

^1^State Key Laboratory of Freshwater Ecology and Biotechnology, Institute of Hydrobiology, Chinese Academy of Sciences, Wuhan 430072, China

^2^Key Laboratory of Environmental and Applied Microbiology, CAS; Environmental Microbiology Key Laboratory of Sichuan Province, Chengdu Institute of Biology, Chinese Academy of Sciences, Chengdu 610041, China

^3^National Fisheries Technical Extension Center, Ministry of Agriculture, P.R. China, Beijing 100125, China

^4^NUMEA, INRA, Univ. Pau & Pays Adour, 64310 Saint Pée sur Nivelle, France

^5^College of Fisheries and Life Science, Shanghai Ocean University, Shanghai 201306, China

^6^Freshwater Aquaculture Collaborative Innovation Center of Hubei Province, Huazhong Agricultural University, Wuhan, Hubei, 430070, China

^*^Corresponding author: Dongyue Feng, Tel: 86-10-59195039, fengdy76@sina.com; Aihua Li, Tel: 86-27-68780053, E-mail address: [liaihua@ihb.ac.cn](mailto:liaihua@ihb.ac.cn)

^§^ These authors contributed equally to this work

**Running title:** Genotype, microbiota and metabolite related

**Supporting Information Methods.**

**Protocols for the extraction and detection of metabolite from gut content samples**

Gut contents samples (about 100mg of fresh weight) stored at -80°C were ground using liquid nitrogen and transferred into 2 mL centrifuge tubes. 1.2 mL of 100% methanol (pre-cooled at -20°C) was then added and vortexed for 10 s, followed by adding 60 µl of ribitol (0.2 mg mL^-1^ stock in deionized water) as an internal quantitative standard and vortexed for 10 s. The tubes were placed into an ultrasound machine at 70℃ for 30 min, and then centrifuged for 10 min at 11,000 g and 1ml supernatant was transferred into 10 ml glass centrifuge tubes. After adding 750ul chloroform (pre-cooled at -20°C) and 1.5 mL deionized water (4°C), the tubes were vortexed for 30 s, then centrifuged for 15 min at 3,000 g. 1 mL supernatant was transferred into a new eppendorf tube. Samples were blow-dried by moderate nitrogen. 50 µl of 15 mg mL^-1^ methoxyamine pyridine solution was then added, vortexed for 30 s and reacted for 90 min at room temperature. Finally, 50 µl BSTFA reagent (containing 1% TMCS) was added into the mixture, reacted for 60 min at 70°C. After the above reactions, samples were determined for contents of metabolites using Agilent 7890A GC system coupled to an Agilent 5975C inert XL EI/CI mass spectrometric detector (MSD) system (Agilent Technologies, Santa Clara, CA, USA). Furthermore, A Mixed n-alkane standard solutions C8–C20 and C21–C40 (Sigma Aldrich) was used for the determination of Retention indices (RI).

1 µL of sample was injected in split mode in a 20:1 split ratio by the auto-sampler. Injection temperature was 280°C, the interface set to 150°C and the ion source adjusted to 230°C. The programs of temperature-rise was followed by initial temperature of 80°C for 5 min, 20°C min^-1^ rate up to 300°C and staying at 300°C for 6 min. Mass spectrometry was determined by full-scan method with range from 35 to 500 (m z^-1^).

For the quality control (QC) samples(Sangster et al., 2006), an aliquot (about 40ul) of all prepared sample extracts, was mixed. These QC samples were used to monitor deviations of the analytical results from these pool mixtures and compare them to the errors caused by the analytical instrument itself. The quality control pool was subsequently divided over 10 vials and analyzed regularly throughout the whole analysis batch. Besides, empty vials referred as “blank controls” were included into the measurement sequence to test for laboratory contaminations.

Data Analysis software and subsequently processed by the XCMS(www.bioconductor.org) using XCMS’s default settings with the following changes: xcmsSet (fwhm = 3, snthresh = 3, mzdiff=0.5, step=0.1, steps=2, max = 300), group (bw = 2, minfrac=0.3, max=300). The signal integration area of each metabolite was normalized to the internal standard (ribitol) for each sample. Identification of metabolites using the Automatic Mass Spectral Deconvolution and Identification System (AMIDS) was searched against commercial available databases such as National Institute of Standards and Technology (NIST) and Wiley libraries. Metabolites were confirmed by comparison of mass spectra and retention indices to the spectra library using a cut-off value of 70 % (Abu Dawud et al., 2012). The XCMS output was further processed using Microsoft Excel (Microsoft, Redmond, WA, USA). Finally, the normalized data were imported into R software for further statistical analyses (Team, 2014).

**References**

Abu Dawud, R., Schreiber, K., Schomburg, D., and Adjaye, J. (2012). Human embryonic stem cells and embryonal carcinoma cells have overlapping and distinct metabolic signatures. *PLoS One* 7(6)**,** e39896. doi: 10.1371/journal.pone.0039896.

Sangster, T., Major, H., Plumb, R., Wilson, A.J., and Wilson, I.D. (2006). A pragmatic and readily implemented quality control strategy for HPLC-MS and GC-MS-based metabonomic analysis. *Analyst* 131(10)**,** 1075-1078. doi: 10.1039/b604498k.

Team, R.C. (2014). *R: A language and environment for statistical computing. R Foundation for Statistical Computing, Vienna, Austria. 2013.* ISBN 3-900051-07-0.

**Table S1.** Genbank accessions of host DNA sequences.

| Species | *cytb* | *COI* |
| --- | --- | --- |
| Grass carp (*Ctenopharyngodon idellus*) | EU391390.1 | EU391390.1 |
| Blunt snout bream (*Megalobrama amblycephala*) | EU434747.1 | EU434747.1 |
| Crucian carp (*Carassius auratus*) | KJ874430.1 | KJ874430.1 |
| Silver carp (*Hypophthalmichthys molitrix*) | EU315941.1 | EU315941.1 |
| Bighead carp (*Hypophthalmichthys nobilis*) | HM162839.1 | HM162839.1 |
| Common rabbits (*Oryctolagus cuniculus*) | [AJ001588.1](https://www.ncbi.nlm.nih.gov/nucleotide/3293006?report=genbank&log$=nuclalign&blast_rank=1&RID=7XV9VAWR015) | AJ001588.1 |

Scientific names are given in parentheses

**Figure S1.** Alpha diversity of gut bacterial community across fish species. Alpha diversity was measured by number of observed OTUs. Groups indicated with the same letter are not significantly different at α = 0.05 using Tukey's post hoc test. Abbreviations: FS, Silver carp; FB, Bighead carp; HG, grass carp; HB, blunt snout bream; OC, crucian carp.


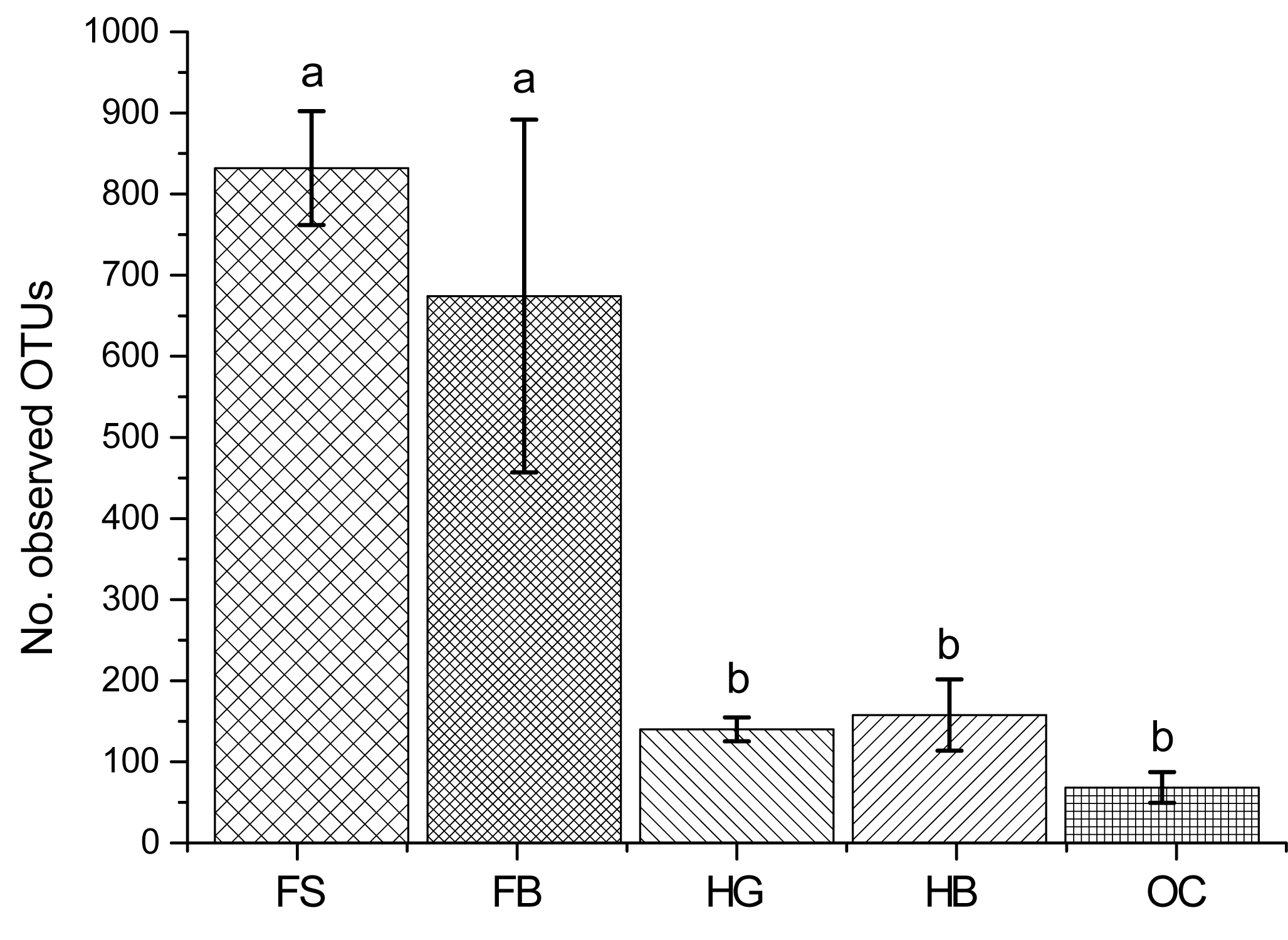


**Figure S2.** The distances of the bacterial communities (A and B) and metabolite profiles (C and D) among the individuals. (A) Distances of the bacterial communities among the individuals between different feeding habits. (B) Distances of the bacterial communities among the individuals between herbivorous and omnivorous species. (C) Distances of the metabolite profiles among the individuals between different feeding habits. (D) Distances of the metabolite profiles among the individuals between herbivorous and omnivorous species. The boxes represent the interquartile range (IQR), from the first and third quartiles, and the central lines represent the median values. Whiskers show 10th and 90th percentiles. Groups indicated with the same letter are not significantly different at α = 0.05 using Tukey's post hoc test. Abbreviations: her, herbivorous; omn, omnivorous; fil, filter-feeding; HG, grass carp; HB, blunt snout bream; OC, crucian carp.


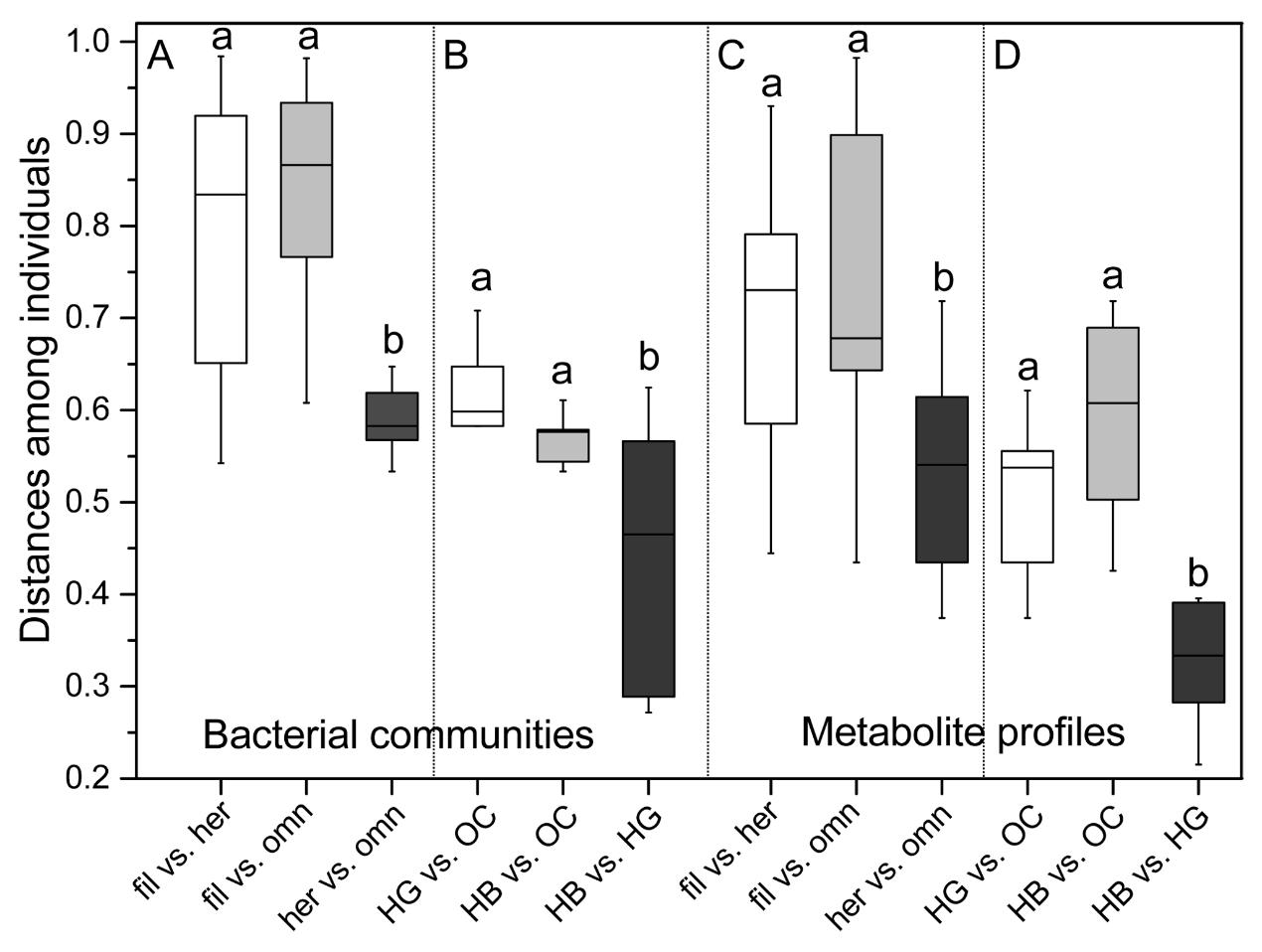


**Figure S3.** Within-group distances of the bacterial communities (A) and metabolite profiles (B) among individuals in different feeding habits. Groups indicated with the same letter are not significantly different at α = 0.05 using Tukey's post hoc test. Abbreviations: her, herbivorous; omn, omnivorous; fil, filter-feeding.


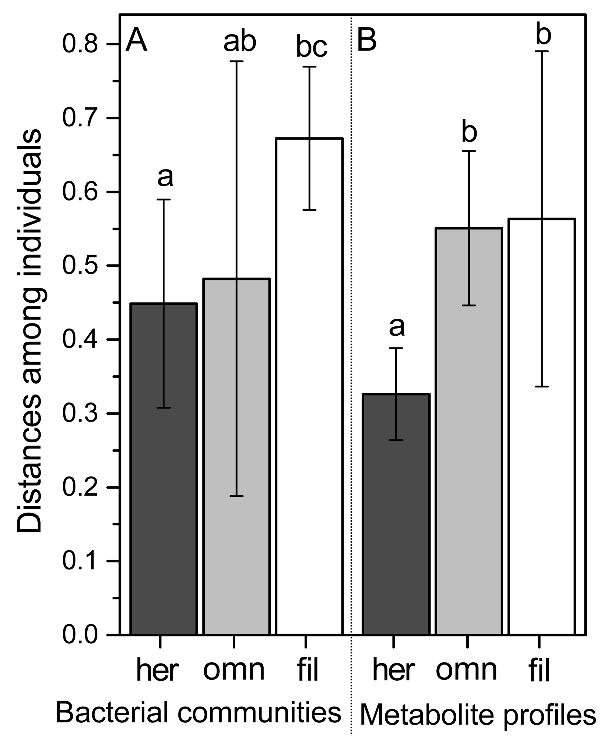


**Figure S4.** PICRUSt classification of KEGG Orthologies (KO). (A) Top 29 level 3 KO functions that differentiate among different fish species as revealed by Random Forest. The other two KEGG pathway hierarchy levels separated by semicolon are also provided. (B) NMDS analysis of level 3 KO functions for different fish species.


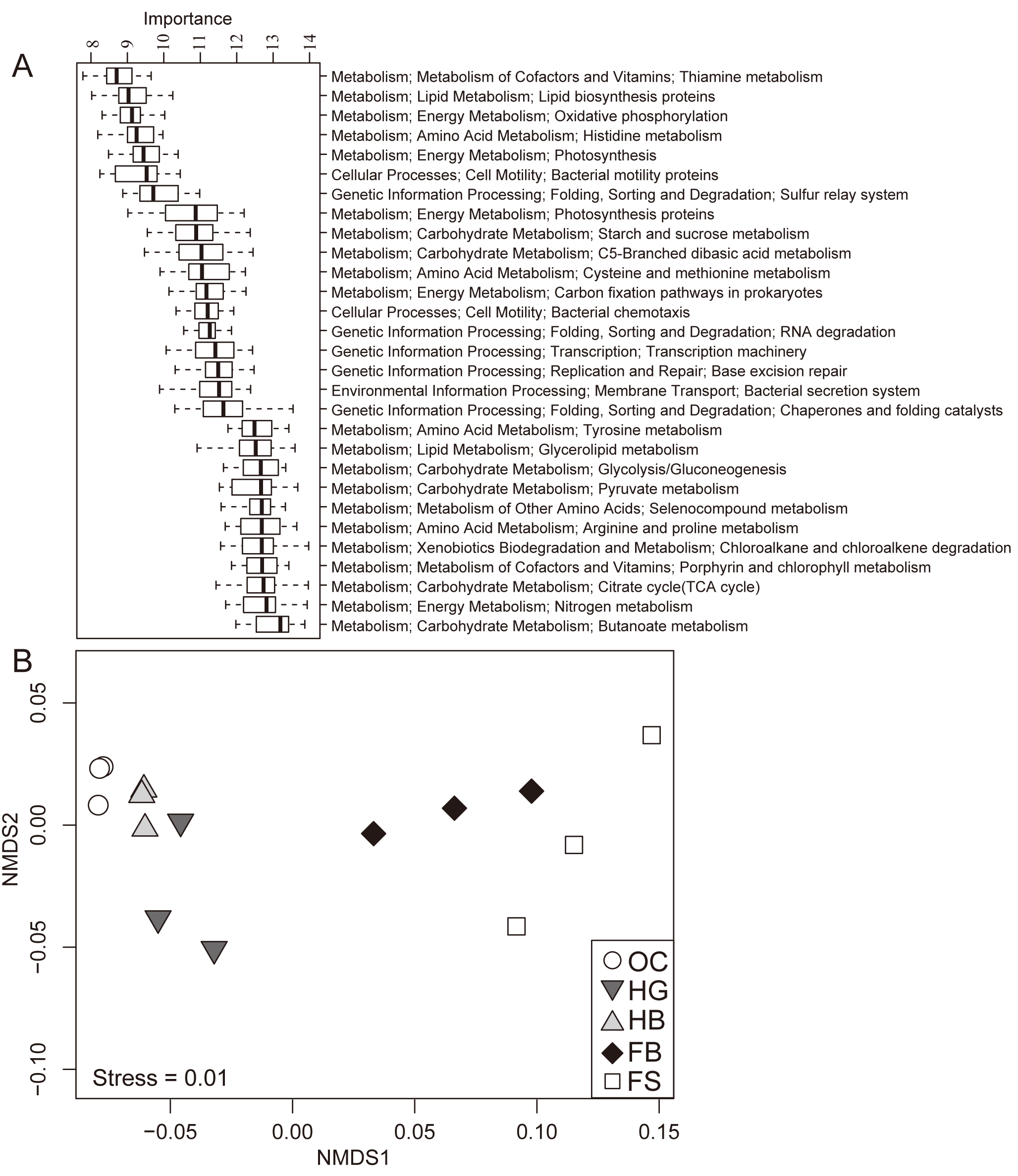

Supplement: Supplementary file 1 [file Data_Sheet_1.docx]
